# Process Optimisation of Ultrasound-Assisted Extraction of Oligosaccharides from Coconut Husk

**DOI:** 10.1155/2023/9427831

**Published:** 2023-04-15

**Authors:** Shie-Lih Tang, Siew-Ling Hii, Chen-Chung Koh

**Affiliations:** Centre for Research of Innovation and Sustainable Development, School of Engineering and Technology, University of Technology Sarawak, No. 1, Jalan Universiti, 96000 Sibu, Sarawak, Malaysia

## Abstract

A fractional factorial design was used to investigate the ultrasound-assisted extraction (UAE) of oligosaccharides from coconut husk, an agroindustry by-product. The effects of five key influencing parameters (*X*_1_, incubation temperature; *X*_2_, extraction duration; *X*_3_, ultrasonicator power; *X*_4_, NaOH concentration; *X*_5_, solid-to-liquid ratio) were studied. Total carbohydrate content (TC), total reducing sugar (TRS), and degree of polymerisation (DP) were the dependent variables. The optimal extraction condition was attained when oligosaccharides with a desired DP of 3.72 were extracted when the coconut husk in a liquid-to-solid ratio of 127 mL/g was treated with 1.05 percent (w/v) of NaOH solution at an incubation temperature of 30.4°C for 5 min using an ultrasonicator power of 248 W. The optimised parameters for oligosaccharide extraction from coconut husk reported in this study could be useful for the effective isolation of these compounds for prebiotic research.

## 1. Introduction

Prebiotics are nondigestible polysaccharides and oligosaccharides that selectively stimulate the growth of beneficial bacteria while inhibiting the growth of pathogenic bacteria. The demand from customers for functional food ingredients and nutraceutical products has dramatically increased in recent years. Therefore, the application of prebiotics as an ingredient in functional foods is an effective marketing strategy when combined with other food products. According to PR Newswire [1], the market for prebiotics is growing significantly, with a growth rate of 11.1%. The prebiotics market is estimated to exceed $6.0 billion in 2022. This has encouraged the food industry to produce more prebiotics or functional oligosaccharides. In general, prebiotics could improve colonic integrity, enhance the immune system, reduce triglyceride levels, control cholesterol levels, enhance mineral absorption, reduce intestinal infections, prevent constipation, inhibit diarrhoea, and suppress allergic responses [2, 3].

Prebiotics can be found in a variety of foods, including onions, garlic, asparagus, chicory root, bananas, seaweed, chocolate, and apples, among others [4]. Sugarcane bagasse, coconut husks, rice husks, empty fruit bunches, oil palm fronds, shells, and fibres, all of which are prevalent agroindustry wastes, could also be employed as viable source materials for extracting the prebiotic. The complex combination of carbohydrate polymers found in these lignocellulosic materials, including cellulose, hemicellulose, and lignin, makes them a useful bioresource for important substances such as prebiotics [5, 6].

Coconut (*Cocos nucifera*) is distributed worldwide in 92 countries, with Indonesia being the largest producer [7]. Malaysia is one of the top ten coconut-producing countries in the world. Coconut is the fourth-most important industrial crop in Malaysia after oil palm, rubber, and rice plantations [8]. The common agricultural wastes that are produced during the harvesting and processing of coconut in the industry are coconut husks, shells, and fronds [9]. These agrowastes could be transformed into value-added products instead of going to direct disposal. The chemical composition of coconut husks includes 38% hemicellulose, 28% cellulose, and 32.8% lignin [10]. The presence of a substantial amount of hemicellulose in coconut husk makes it a potential substrate that could be used for the production of oligosaccharides with prebiotic properties. Nevertheless, there are many obstacles to overcome in order to utilise the lignocellulosic materials effectively. The extraction process plays a crucial role in the efficient recovery of bioactive components such as oils, proteins, and polysaccharides from these resources for their application or for further research [11].

Conventional procedures for extracting polysaccharides from plant tissue typically include the use of solvents or chemicals such as water, acids, or alkalis at high temperatures, which requires a prolonged processing period [12]. Due to the low extraction yield and efficiency of extracted polysaccharides, these conventional extraction methods have very few practical applications [13]. As an alternative to traditional methods, the ultrasonic system has recently been widely adopted. Ultrasound in the range of 20 kHz to 2000 kHz is used in the UAE. The acoustic cavitation of ultrasound can increase the surface contact between solvents and samples as well as the permeability of cell walls [14, 15]. Acoustic cavitation refers to the bubble formation, growth, and implosion during the propagation of an ultrasound wave in the solvent [16]. There are two types of devices that can supply high-power ultrasound, namely, ultrasonic baths and probe-type ultrasound equipment. For both systems, a transducer acts as the source of ultrasound power. The ultrasonic bath is the most common device, which includes a stainless-steel tank and transducers [17].

The basis of ultrasound, sonochemistry, has frequently been considered a “clean technology” that has sparked interest in recent years in view of its numerous advantages over traditional approaches. In the food sector, ultrasound technology provides a nonthermal processing option. Processes including viscosity modification, functionality enhancement, and nutrient delivery could be effectively accomplished in the food industry by subjecting food ingredients to ultrasonic treatment. UAE is also an energy-efficient technique since it provides great extraction yields due to its selectivity, energy savings due to its brief operating time, and cost-effectiveness because it only requires a little amount of solvent for extraction, resulting in less waste being produced [18, 19].

Among the few advantages of using the UAE to extract polysaccharides, as cited by Chemat et al. [20], were improved mixing, quicker heat and mass transfer, smaller equipment, quicker start-up, and a higher extraction yield. In a study conducted by Jovanovic-Malinovska et al. [21], UAE was used to extract prebiotics from four types of fruits and eight types of vegetables under optimal conditions of 63% v/v ethanol, 40°C temperature, and a 10-minute extraction time. Their research revealed that the specific oligosaccharide fractions obtained by UAE varied depending on the type of raw materials employed. Additionally, compared to the conventional method, the sonication process increased total oligosaccharides by a factor of 2 to 4.

Nevertheless, integrating this technology into large-scale industrial processes is still a difficult task. One of the main issues with the scaling up of the ultrasound process in industrial processes is the lack of large-scale ultrasonic reactors. The cost of implementing this technology in companies is now higher because ultrasonic equipment must still be designed specifically for each application. Additionally, in some applications, it is found to be difficult to intensify bubble formation and distribute acoustic energy evenly throughout the extraction solution. Despite these difficulties in constructing large-scale equipment at the moment, some sectors, including the dairy industry and other food processing sectors, are gradually looking into the possibility of integrating this technology into their processing facilities. When the various industrial sectors recognise the potential benefits of this technology, the obstacles may be overcome.

In the present study, a fractional factorial design (FrFD) followed by augmented response surface methodology (RSM) was utilised to optimise the UAE of oligosaccharides from coconut husk. A factorial design is best for removing nonstatistically significant independent variables [22]. In addition, FrFD has been recommended to be used when there are many factors, around 5 factors, to be studied [22]. The RSM then helps researchers enhance process conditions, evaluate the effects of variables on products, and optimise process conditions, ultimately leading to an enhanced process or better-quality product [23]. The factors can be examined simultaneously using these statistical experimental designs, and the potential interactions between the factors can then be classified [24]. Furthermore, the statistical experimental design enables the investigation of potential combinations of experimental runs under the constraints of limited resources, including the cost of the experiment [22].

RSM has been used to improve the yield of oligosaccharides and polysaccharides from lotus seeds [25], Chinese traditional herbal medicine *Angelica sinensis* [26], straw mushrooms [27], and sweet potatoes [18]. On the other hand, the reports on the effects of process variables that aim for the elevated level of TC and the desired DP have been very limited. The goal of the current study was thus to optimise the UAE conditions on the TC level and DP of the coconut husk extract.

Five influential operating parameters on the UAE process were initially screened using an FrFD. Following the identification of the curvature, the response model was developed by augmenting the FrFD experimental design to the RSM. Higher-order terms were generated using augmented RSM to explain the curvature. In order to account for curvature, more star points were added [28]. This allows the experimenter to save time by not having to build another RSM model from scratch. The validation of optimal UAE conditions for extracting oligosaccharides with the desired DP was then conducted.

## 2. Materials and Methods

### 2.1. Collection and Processing of Sample

The coconut husk was cleaned thoroughly under running tap water after collection from the local market. The coconut husk was dried in an oven dryer until constant weight and then grinded into fine powder. The powder was sieved to pass through a 250 *µ*m mesh sieve and stored in a container at ambient temperature until further analysis.

### 2.2. Two-Level Fractional Factorial Design (FrFD)

The FrFD with five independent variables (*X*_1_, incubation temperature; *X*_2_, duration of extraction; *X*_3_, power of ultrasonicator; *X*_4_, NaOH concentration; *X*_5_, solid-to-liquid ratio) at two levels was first used to explore and optimise the effects of these independent variables on the contents of total carbohydrates (*Y*_1_) and total reducing sugar (*Y*_2_) as well as the degree of polymerisation (*Y*_3_) of the extract. Each independent variable was assigned two levels, i.e., low level (−1) and high level (+1), which corresponded to the minimum and maximum values of the factors under investigation. In addition, three centre-point experiments at the mean level (0) were added to estimate the variation of the model. The experimental factors and the actual levels are illustrated in Table 1.

The 2^5 − 1^ two-level FrFD is a design with five factors that consists of eight different combinations, two blocks, and three centre-point experiments for a total of 22 experimental runs (Table 2). These experimental runs were conducted in duplicate. Uncoded (actual) values are shown in the brackets with the coded values at the low, mean, and high levels.

### 2.3. Augmented Response Surface Methodology (RSM) Design

Following the fractional factorial design of experimental works, an augmented RSM was performed with additional experimental runs (Runs 23 to 34 shown in Table 2) around the centre point of the previous design matrix.

### 2.4. Extraction and Precipitation Procedures

The coconut husk powder was mixed with a NaOH solution based on the design generated by the software (Table 2). The mixture was then subjected to ultrasound extraction with the respective setting. After that, the mixture was centrifuged at 4°C at 4500 rpm for 10 min. The resulting supernatant was then subjected to an overnight precipitation process with three volumes of cold ethanol. Subsequently, the mixture solution was centrifuged again at 4°C at 4500 rpm for 10 min to isolate the precipitate. The precipitate was collected and dried at 60°C until constant weight. The total carbohydrates (*Y*_1_), total reducing sugar (*Y*_2_), and degree of polymerisation (*Y*_3_) of the extract were then determined. All the processes were conducted in duplicate.

### 2.5. Statistical Analysis of FrFD and RSM

The collected data were analysed with the software Design-Expert Version 10.0 (Stat-East, Inc.). The normality of the data was checked before the interpretation of the models. ANOVA was used to calculate the *F*-values and *p* values of all the model terms. Models with *p*-values less than 0.05 are considered statistically significant. Following the regression analysis and ANOVA, model equations were produced to predict and optimise the UAE of oligosaccharides from coconut husk.

### 2.6. Validation of Response Model for Oligosaccharide Extraction

To validate the reliability of the response surface model, a verification process was performed based on the desirability value generated by the software. Numerical optimisation was carried out using the technique of Derringer's desirability function. This study aims to produce prebiotics with a maximum level of TC content while keeping the DP value within the range of 3 to 8. The optimum condition with the desirability nearest to one was chosen to be validated. All the experimental runs were then performed in triplicate to validate the optimised extraction conditions.

### 2.7. Analytical Procedures

#### 2.7.1. Determination of TC

Phenol-sulphuric acid assay was used to identify the total concentration of carbohydrates in the extract by using glucose as a standard sugar solution [29]. One mL of the extract was mixed with 1 mL of the 5% phenol solution and 5 mL of concentrated sulphuric acid. The mixture was then vortexed and incubated in a 30°C water bath for 30 minutes. After that, the absorbance of the mixture was measured at 490 nm.

#### 2.7.2. Determination of TRS

The concentration of reducing sugar was determined by the dinitrosalicylic acid (DNS) method [30]. One millilitre of the extract was mixed with 1 mL of DNS reagent, followed by two drops of 10% sodium hydroxide, and boiled for 15 minutes. The mixture was then cooled under running tap water. Ten millilitres of distilled water were then added and vortexed. The mixture was left for 20 minutes, and the absorbance was measured at 540 nm.

#### 2.7.3. DP

The degree of polymerisation of the oligosaccharides was calculated using the following equation [31]:

(1) $$\begin{array}{c} degree\;of\;polymerisation\;\left(DP\right)=\frac{total\;carbohydrates}{total\;reducing\;sugar}\text{.} \end{array}$$

## 3. Results and Discussion

### 3.1. FrFD and Augmented Response Surface Methodology (RSM) Design

The two-level FrFD was the first experimental design employed in this study to assess the impact of parameters on responses. The extracted oligosaccharide from the experimental runs was analysed on the basis of the three responses. The normality of the experimental data was initially confirmed after all the tests were completed. Model *p* values, curvature and lack of fit, adjusted coefficient of determination (adj-*R*^2^), predicted coefficient of determination (pred-*R*^2^), percentage of coefficient of variation (% CV), acceptable accuracy, and projected residual sum of square (PRESS) were then examined. Following the ANOVA analysis of FrFD, augmented RSM is a continuation of that method for resolving the problem with the significant curvature generated by the factors (with a *p*-value of 0.005) and the response variable. The experimenter can save time by using this continuation of FrFD rather than starting over with another RSM model.

#### 3.1.1. Response *Y*_1_

*TC.* The model of FrFD for Response *Y*_1_ (TC) is inverse square root transformed. In its original scale, the Response *Y*_1_ was found to give an S-shaped curve, indicating the deviation of the data from the normal range (Figure S1 in the supplementary materials). The transformation was thus required in an attempt to accommodate the experimental data so that it was in the range of normality and homogeneity [32]. This transformation yielded a Shapiro W value close to one (*W* = 0.954), implying normality [33]. The normality assumption for all the response models was evaluated after the transformation. Linearity of all the experimental data was also assessed.

The overall transformed model for Response *Y*_1_ was highly significant with a model *p*-value <0.001. The lack of fit for Response *Y*_1_ was found to be insignificant with a *p*- value of 0.580. However, the significant curvature was detected with a *p*-value of 0.005, which is less than 0.05 (Table S1 in the supplementary materials). This finding suggests that the two-level FrFD model of Response *Y*_1_ model adequacy checking is insufficient for capturing the true link between the input components and Response *Y*_1_ [34]. Therefore, it is necessary to replace the current two-level FrFD design with one that can accurately explain the experimental data. The second-order response surface model, which offers protection against curvature, is a logical model that may be used to fit the experimental data. As a result, the current design was then augmented to RSM, which could be used to estimate the curvature [27, 34]. In the design matrix of the augmented RSM, several additional runs around the centre point for the curvature estimation were carried out (Table 2; combinations 19 to 30).

The generated model for Response *Y*_1_ of the augmented RSM study was found to be significant with an *F*-value of 16.77 and *p* < 0.001 (Table S2 in the supplementary materials). The close relationship between the model and the data could be confirmed when the *R*^2^ value was closer to one (Table 3). The close agreement within 0.2 between adjusted-*R*^2^ (0.8207) and predicted-*R*^2^ (0.7055) of Response *Y*_1_ suggested that the fitted model was capable of predicting future responses [35, 36]. An adequate precision of 15.1859 indicates that the model can be used to navigate the design space [37, 38]. Furthermore, the small PRESS (sum of the squares of all the resulting prediction errors) value of this response model (PRESS = 0.0098) indicates negligible error in this model [39].

Following the augmented RSM investigation, two types of mathematical equations—coded equations and actual equations—were established. Predictions about the influence of various factors on Response *Y*_1_ are made using the equation in terms of coded factors (equation (2)) [40]. It is not recommended to use the actual term equation to calculate the relative impact of each factor because the coefficients are scaled to accommodate the units of each factor and the intercept is not at the centre of the design space [41].

Coded equation for Response *Y*_1_:

(2) $$\begin{array}{c} \frac{1}{\sqrt{Y_{1}}}\left(\times 10^{-4}\right)=1377.5–92.11X_{1}–87.81\;X_{2}+6.59\;X_{3}–335.64\;X_{4}+185.42\;X_{5}–88.25\;X_{1}X_{3}+120.43\;X_{2}X_{4}+85.58\;X_{3}X_{4}+290.34\;X_{2}\text{.} \end{array}$$

The correlation effects of the individual terms were analysed on the basis of the mathematical sign (plus (+) or minus (−)). The plus (+) sign indicates a positive effect while the minus (−) sign indicates a negative effect on the model term [34]. However, because the response was transformed into an inverse square root model, the correlation effects of the response model component were in the opposite direction. Plus (+) here denotes a negative effect, while minus (−) denotes a positive effect. Three single variables—temperature (*X*_1_), time (*X*_2_), and sodium hydroxide concentration (*X*_4_)—as well as variables with a two-way interaction between temperature and power (*X*_1_*X*_3_), all had a positive impact on Response *Y*_1_. Additionally, *X*_4_ had the greatest positive impact on *Y*_1_ due to its highest coefficient (335.64 × 10^−4^) while the main effect of the solid-to-liquid ratio (*X*_5_) was the most significant negative model term.

In the present study, the main effect of NaOH concentration (*X*_4_) was the most significant positive model term with a *F*-value of 75.60 (Table 4). It has been reported that sodium hydroxide can dissolve the hemicellulose and break the linkage of lignin with cellulose [42, 43]. The cellulose and hemicellulose were further transformed into oligo- or disaccharides [44]. However, as shown by the negative correlation effects between these variables, it is advised to utilise lower sonication power (*X*_3_) or shorter extraction duration (*X*_2_) in combination with a higher NaOH concentration (*X*_4_), or vice versa, in order to extract higher levels of total carbohydrate content (Table 4). Longer solvent extraction duration for samples treated with highly concentrated solvent was found to seriously damage the sample's structure [45].

The main effect of the solid-to-liquid ratio (*X*_5_) had the most detrimental impact (negative sign of correlation effect with *F*-value of 26.07) on the amount of total carbohydrates extracted, which suggests that the amount of total carbohydrates recovered was lower when more coconut husk was used in the extraction process. Coconut husk, the solute in the current work, may become supersaturated in the extraction solvent when a very high solid-to-liquid ratio was applied. On the other hand, the mass transfer dynamics of oligosaccharides and solvent improved when a higher volume of solvent was used [46]. The divergence of solvent within and outside samples will increase with a higher volume of solvent added to the mixture. These phenomena boost energy absorption and expand the sample's surface area in contact with the solvent. When this happened, the oligosaccharides would be discharged into the medium outside the cell [46, 47]. However, an increase in solvent consumption could also result in an increase in production costs [48]; and an increase in solvent volume does not necessarily translate into a bigger concentration difference in the yield of polysaccharides [49]. In light of this, it was not necessary to increase the volume of the solvent used for the present study, which results in solvent waste consumption.

The current investigation showed that the extraction temperature (*X*_1_) had a favourable impact on the total amount of extracted carbohydrates (*Y*_1_). The oligosaccharides in the plant matrix were able to be mobilised by adjusting the temperature throughout the extraction process [48]. The extraction of bioactive compounds, such as prebiotics, is greatly influenced by temperature. However, the degradation of compounds may occur concurrently with temperature increases above a particular threshold. In addition, as temperatures rise, solvent loss from vaporisation may speed up, increasing the expense of the extraction process [21].

In addition to the distinct main effects of each individual factor, there were significant second-order quadratic interactions and two-way interactions of the input factors. With an *F*-value of 6.00, it can be seen that the two-way interaction between temperature and power (*X*_1_*X*_3_) had a positive impact on the total carbohydrate content (Table 4). The cavitation capacity of the ultrasonication process depends on a number of factors, including the frequency of the ultrasonicator and the viscosity and surface tension of the product, as well as the temperature and pressure of the surrounding environment [50]. The cavitation produced by an ultrasonicator with increasing power can easily penetrate liquid media [51]. The high power of the ultrasonicator also enables the solvent molecules that produce the bubbles to migrate and rupture on the sample's surface. This situation accelerates the release of solute (in this case, cellulose and hemicellulose) from the sample before it can be broken down into oligosaccharides [52]. Therefore, it is essential to have both the temperature and power at a certain ideal setting in order to produce the extract with a high amount of total carbohydrates.

#### 3.1.2. Response *Y*_2_: TRS

To account for the data's normality and homogeneity, the model for Response *Y*_2_ was also inverse square root transformed. The model was implied to be significant by the model's *F*-value of 25.46 and *p*-value 0.001. A *p*-value of 0.531 indicated that the lack of fit for Response *Y*_2_ was not significant. It was possible to assume that Response *Y*_2_ was linear based on the obtained insignificant *p*-value of curvature (*p* = 0.319). This demonstrated that the two-level fractional factorial design was adequate for this set of data (Table S3 in the supplementary materials). The difference between adjusted-*R*^2^ (0.8374) and predicted-*R*^2^ (0.7156) was less than 0.2, indicating that the model was fit (Table 3).

The model term *X*_5_ with the greatest *F*-value had the most significant negative impact on the TRS, as shown in Table 5. This means that the amount of reducing sugar decreased when a higher solid-to-liquid ratio was used in the reaction mixture. As shown in equation (1), the degree of polymerisation is determined by dividing the total amount of carbohydrate by the total amount of reducing sugar. As a result, the extract with a low total reducing sugar content is preferred because it likely contains a longer oligosaccharide chain or a higher DP value [53].

With an *F*-value of 14.85, the extraction time (*X*_2_) was the most significant positive factor for the extract's TRS concentration (Table 5). The concentration of reducing sugar increased as the extraction process took longer. Oligosaccharide disintegration was accelerated by the build-up of heat energy from sonication [25]. Additionally, a longer extraction period may cause polysaccharides to degrade further [47]. As a result, the mixture will yield more monomeric sugars.

The interaction between sodium hydroxide concentration (*X*_4_) and ultrasonicator power (*X*_3_) had a less pronounced negative effect with an *F*-value of 4.91 (Table 5). The interaction of these two factors had a detrimental impact on the extract's total reducing sugar content. That said, while using a lower concentration of sodium hydroxide (*X*_4_) and increasing the sonication power (*X*_3_), the concentration of total reducing sugar in the coconut husk extract increased, and vice versa. Harsh extraction conditions, such as using an ultrasonicator with a high power and a high sodium hydroxide concentration, could cause the oligosaccharides in the extract to lose their structural integrity [47]. The model terms that have a negative impact on the TRS are preferred for Response *Y*_2_, since this indicates that the majority of oligosaccharides were not converted to monomeric sugar form.

There was an interesting interaction between the power (*X*_3_), sodium hydroxide concentration (*X*_4_), and temperature (*X*_1_). This three-way interaction's correlation effect was positively significant with an *F*-value of 7.87 (Table 5). More monomeric sugars were found when the temperature, ultrasonic power, and sodium hydroxide concentration were all high. The occurrence was probably caused by the degradation of oligosaccharides into reducing sugar under the exceedingly harsh extraction conditions, which were not ideal in the current study.

#### 3.1.3. Response *Y*_3_

*DP.* The inverse square root transformed model for Response *Y*_3_ was a model with *p*-value <0.001 and insignificant lack of fit (*p*-value = 0.568). The significant *p*-value of curvature (*p*-value = 0.004), however, made it impossible to conclude that Response *Y*_3_ is linear (Table S4 in the supplementary materials). In order to explain the considerable curvature that emerged from the experimental data, the initial two-level FrFD was modified into an augmented RSM optimal with additional runs around the centre point (Table 2). Following the modification of the design matrix, the validity of the model constructed with augmented RSM was confirmed with a significant model (*p*-value <0.001) and insignificant lack of fit (*p*-value = 0.735), indicating the model is fit. The model adequacy was checked using similar procedures as previous responses (Table S5 in the supplementary materials). Model terms with a *p*-value less than 0.05 indicated that the model terms were significant. Similar to the analysis of responses *Y*_1_ and *Y*_2_ mentioned previously, the significant factors affecting response *Y*_3_ were able to be identified by examining the *p*-value of each of the model terms.

The most significant positive model term, with an *F*-value of 27.00, was the main effect of sodium hydroxide concentration (*X*_4_) (Table 6). The findings suggested that using higher concentrations of sodium hydroxide during extraction would produce oligosaccharide extracts with higher DP values. The solid-to-liquid ratio (*X*_5_), another significant positive model term with an *F*-value of 23.49, indicated that when the coconut husk was supersaturated in the extraction solvent, the majority of the husk was left in the complex structure form. Therefore, it is important to adopt the appropriate solid-to-liquid ratio throughout the extraction process in order to obtain oligosaccharides with the desired DP values.

With an *F*-value of 5.82, the negative model term of the individual effect of extraction time (*X*_2_) showed that extracts with lower DP values were produced when longer extraction times were used. The negative relationship between extraction duration (*X*_2_) and sodium hydroxide concentration (*X*_4_) also suggested that a longer extraction duration and a higher solvent concentration would result in an extract with a simpler structure and, thus, a lower DP value. This occurrence was probably caused by the extensive breakdown of the coconut husk structure during treatment, which was caused by the prolonged extraction duration and high solvent concentration discussed earlier. When a prolonged sonication duration was used to extract polysaccharides, Sun and Tomkinson [54] found similar results: the molecular weight of the polysaccharides increased. However, extending the extraction duration further led to the breakdown of the b-O-4 linkage, which in turn reduced the molecular weight of the polysaccharides. Therefore, determining the optimal and appropriate extraction duration is crucial since prolonged extraction may result in polysaccharide hydrolysis and disintegration [47].

The interaction between the solid-to-liquid ratio (*X*_5_) and ultrasonicator power (*X*_3_), which had an *F*-value of 4.38, was another less obvious positive correlation impact. When a sample with a higher solid-to-liquid ratio was employed in the current study, an ultrasonicator with a power range of 115 W to 205 W could produce oligosaccharide with a DP value of 3 to 8. According to a study by Lu et al. [25], the positive interaction effect of ultrasonic power and the solid-to-liquid ratio boosted the oligosaccharide yield. However, when greater ultrasonicator power and a higher solid-to-liquid ratio were used in the extraction process, the yield of oligosaccharides dropped, according to their findings. Because of this, the solid-to-liquid ratio and ultrasonicator power employed in the subsequent validation test in the current investigation were restricted to 1 : 50 to 1 : 150 and 25 to 250 W, respectively.

### 3.2. Validation of Optimised Extraction Conditions

An additional set of experiments was conducted in triplicates to verify the experiment's model (Table 7). Under the solution with the best desirability (equal to one), the factor levels were set at the values forecasted by the software. In this study, the greatest *D* value attained is 1. A greater desirability value (*D*) denotes that the experimental outcomes are more likely to meet the goal (predicted responses).

In comparison to the predicted value of 13.77 mg/mL, the experimental data for TC were 14.29 mg/mL (Table 7). Additionally, an extract with 3.84 mg/mL of TRS and DP of 3.72 was produced under the suggested optimal extraction conditions. It is preferred to have lower TRS concentrations with greater DP values compared to the expected values. Since the actual and projected values were in close agreement, the validation results revealed that the response model could accurately reflect the optimisation of oligosaccharide extraction from coconut husk.

In comparison to the published literature, the current optimisation study successfully observed lower extraction temperatures (30.4°C) and sonication power (248.0 W) with a shorter extraction duration (5 min). For the UAE of oligosaccharides from diverse plant or fruit sources, for instance, Lu et al. [25] reported 5.42 min and 300.5 W, Wu et al. [48] reported 51.5°C, 20 min, and 440 W, Jovanovic-Malinovska et al. [21] reported 40.0°C and 10 min, and Maran et al. [51] reported 56.0°C and 17 min. In terms of extract yield, the current study was able to extract more oligosaccharides (15.5% g/g) from coconut husk than most studies that have previously been published, including 4.33% (g/100 g) from pumpkin by Wu et al. [48], 6.22% (g/100 g) from scallion by Jovanovic-Malinovska et al. [21] and 6.06% (g/g) from corn silk by Maran et al. [47].

## 4. Conclusions

In this study, the two-level FrFD and augmented RSM were found to be useful in constructing mathematical equations and model graphs for the optimal operating conditions of the UAE that affect the amount of TC and TRS extracted from coconut husk as well as the DP of the extract. An extract with a DP of about 4.00 was produced when 1.05% (w/v) NaOH solution was applied to coconut husk with a solid-to-liquid ratio of 1 : 127 in an ultrasonicator running at 248 W for 5 minutes at 30.4°C. The three responses' experimental values (*Y*_1_, *Y*_2_, and *Y*_3_) closely match those predicted by the equations. This demonstrated that the overall error was small, demonstrating the model's applicability for optimisation.

## Figures and Tables

**Table 1:** Experimental factors and corresponding levels used.

Notation	Factor	Unit	Low	Mean	High
*X*_1_	Extraction temperature	°C	30	40	50
*X*_2_	Extraction time	min	5	25	45
*X*_3_	Ultrasonicator power	Watt	25	137.5	250
*X*_4_	Sodium hydroxide concentration	% w/v	1	6.5	12
*X*_5_	Solid-to-liquid ratio	—	50	100	150

**Table 2:** Experimental design matrix FrFD (run 1 to run 22) and augmented RSM (run 23 to run 34) and responses.

Run	Experimental factors^*a*^					Responses^*b*^		
	*X*_1_	*X*_2_	*X*_3_	*X*_4_	*X*_5_	*Y*_1_	*Y*_2_	*Y*_3_
1	+1 (50)	+1 (45)	+1 (250)	−1 (1)	+1 (150)	31.58	7.31	4.32
2	+1 (50)	−1 (5)	+1 (250)	+1 (12)	−1 (50)	83.16	16.61	5.01
3	−1 (30)	+1 (45)	+1 (250)	+1 (12)	−1 (50)	52	19.85	2.62
4	0 (40)	0 (25)	0 (137.5)	0 (6.5)	0 (100)	38.82	11.56	3.36
5	−1 (30)	−1 (5)	−1 (25)	−1 (1)	−1 (50)	19.9	11	1.81
6	−1 (30)	−1 (5)	+1 (250)	−1 (1)	+1 (150)	13.78	6.66	2.07
7	0 (40)	0 (25)	0 (137.5)	0 (6.5)	0 (100)	40.64	12.3	3.3
8	0 (40)	0 (25)	0 (137.5)	0 (6.5)	0 (100)	40.64	12.3	3.3
9	+1 (50)	−1 (5)	−1 (25)	+1 (12)	+1 (150)	37.79	4.97	7.61
10	+1 (50)	+1 (45)	−1 (25)	−1 (1)	−1 (50)	39.26	29.62	1.33
11	−1 (30)	+1 (45)	−1 (25)	+1 (12)	+1 (150)	45.39	21.48	2.11
12	+1 (50)	−1 (5)	+1 (250)	−1 (1)	−1 (50)	29.11	21.32	1.37
13	+1 (50)	−1 (5)	−1 (25)	−1 (1)	+1 (150)	14.05	5.71	3.84
14	0 (40)	0 (25)	0 (137.5)	0 (6.5)	0 (100)	64.93	10.11	6.42
15	−1 (30)	+1 (45)	+1 (250)	−1 (1)	−1 (50)	31.43	28.33	1.11
16	−1 (30)	−1 (5)	−1 (25)	+1 (12)	−1 (50)	88.59	15.29	5.79
17	−1 (30)	−1 (5)	+1 (250)	+1 (12)	+1 (150)	39.15	4.73	8.28
18	+1 (50)	+1 (45)	−1 (25)	+1 (12)	−1 (50)	68.41	22.86	2.99
19	+1 (50)	+1 (45)	+1 (250)	+1 (12)	+1 (150)	33.03	8	4.13
20	−1 (30)	+1 (45)	−1 (25)	−1 (1)	+1 (150)	20.06	5.48	3.66
21	0 (40)	0 (25)	0 (137.5)	0 (6.5)	0 (100)	43.77	7.99	5.44
22	0 (40)	0 (25)	0 (137.5)	0 (6.5)	0 (100)	37.64	9.2	4.09
23	−1 (30)	−1 (5)	+1 (250)	0 (6.5)	−1 (50)	29.48	11.65	2.53
24	−1 (30)	+1 (45)	+1 (250)	0.333 (8.3)	+1 (150)	36.05	3.72	9.68
25	0 (40)	−1 (5)	+1 (250)	+1 (12)	−1 (50)	81.71	14.2	5.75
26	+1 (50)	−0.333 (18.3)	−1 (25)	−1 (1)	−0.333 (83.3)	34.82	11.55	3.01
27	−1 (30)	+1 (45)	−0.33 (100)	−1 (1)	−0.333 (83.3)	30.45	7.75	3.93
28	+1 (50)	−1 (5)	+1 (250)	−0.333 (4.7)	+1 (150)	42.36	5.12	8.26
29	+1 (50)	+1 (45)	+1 (250)	0.333 (8.3)	−0.333 (83.3)	74.56	19.45	3.83
30	+1 (50)	−1 (5)	−0.333 (100)	−0.333 (4.7)	−1 (50)	76.81	15.45	4.97
31	+1 (50)	−0.333 (18.3)	−1 (25)	−1 (1)	−0.333 (83.3)	34.57	12.37	2.79
32	0 (40)	−1 (5)	+1 (250)	+1 (12)	−1 (50)	76.79	11.73	6.54
33	−1 (30)	−0.333 (18.3)	−0.333 (100)	−1 (1)	+1 (150)	33.54	4.84	6.94
34	−1 (30)	+0.333 (31.7)	−1 (25)	−0.333 (4.7)	−1 (50)	78.84	14.18	5.56

^*a*^Factors: *X*_1_, temperature (°C); *X*_2_, time (min); *X*_3_, power (watt); *X*_4_, sodium hydroxide concentration (%w/v); *X*_5_, solid-to-liquid ratio. Uncoded values are presented in the brackets. ^*b*^Responses: *Y*_1_, TC (mg/mL); *Y*_2_, TRS (mg/mL); *Y*_3_, DP.

**Table 3:** Regression analysis of variance for all three responses.

Descriptors	Responses		
	TC (*Y*_1_)	TRS (*Y*_2_)	DP (*Y*_3_)
Mean	0.1606	0.3115	0.3521
Standard deviation	0.0173	0.0365	0.0256
Percentage of coefficient of variation (%C.V.)	10.76	11.71	7.26
*R*-squared (*R*^2^)	0.8728	0.8716	0.7400
Adjusted *R*-squared (adj-*R*^2^)	0.8207	0.8374	0.6776
Predicted *R*-squared (pred-*R*^2^)	0.7055	0.7156	0.5210
Adequate precision	15.1859	15.5013	12.8502
PRESS	0.0152	0.0442	0.0301

**Table 4:** *F*-value, *p* value, and the correlation effect of the augmented RSM model terms on Response *Y*_1_.

Model terms	*F*-value	*p* value	Correlation effect
*X*_1_	7.22	0.014^*∗*^	+
*X*_2_	6.00	0.023^*∗*^	+
*X*_3_	0.0318	0.860	−
*X*_4_	75.60	<0.001^*∗*^	+
*X*_5_	26.07	<0.001^*∗*^	−
*X*_1_ * X*_3_	6.00	0.023^*∗*^	+
*X*_2_ * X*_4_	9.19	0.006^*∗*^	−
*X*_3_ * X*_4_	4.37	0.048^*∗*^	−
*X*_2_^2^	16.41	0.001^*∗*^	−

^*∗*^ *p* < 0.05 is significantly different.

**Table 5:** *F*-value, *p* value, and correlation effect of the model terms on Response *Y*_2_.

Model terms	*F*-value	*p* value	Correlation effect
*X*_2_	14.85	0.002^*∗*^	+
*X*_5_	74.21	<0.001^*∗*^	−
*X*_3_ * X*_4_	4.91	0.043^*∗*^	−
*X*_1_ * X*_3_ * X*_4_	7.87	0.013^*∗*^	+

^*∗*^ *p* < 0.05 is significantly different.

**Table 6:** *F*-value, *p* value, and correlation effect of the model terms on Response *Y*_3_.

Model terms	*F*-value	*p* value	Correlation effect
*X*_2_	5.82	0.023	−
*X*_4_	27.00	<0.001	+
*X*_5_	23.49	<0.001	+
*X*_2_ * X*_4_	9.35	0.005	−
*X*_3_ * X*_5_	4.38	0.047	+
*X*_3_^2^	9.72	0.005	−

^*∗*^ *p* < 0.05 is significantly different.

**Table 7:** Input factors, predicted responses, and experimental data of the optimised formulation with the highest desirability function.

Combination	Notation	Value
Factors^*a*^	*X*_1_	30.39
	*X*_2_	5.02
	*X*_3_	248.08
	*X*_4_	1.05
	*X*_5_	127.01

Predicted responses^*b*^	*Y*_1_	13.77
	*Y*_2_	6.41
	*Y*_3_	3.21

Experimental data^*c*^	*Y*_1_	14.29 ± 0.55
	*Y*_2_	3.84 ± 0.05
	*Y*_3_	3.72 ± 0.18

^*a*^Predicted optimised extraction conditions by the software. ^*b*^Predicted values of responses. ^*c*^Experimental data (in average ± standard deviation, *n* = 3).

## Data Availability

The data (Figure S1 and Tables S1–S5) used to support the findings of this study are included in the supplementary materials.

## Supplementary Materials

Supplementary Materials Figure S1. Normality plot of residuals before transformation. Table S1. ANOVA test for total carbohydrates (Y1) by two-level fractional factorial design. Table S2. ANOVA test for total carbohydrates (*Y*1) by augmented RSM. Table S3. ANOVA test for total reducing sugar (*Y*2) by two-level fractional factorial design. Table S4. ANOVA test for the degree of polymerisation (*Y*3) by two-level fractional factorial design. Table S5. ANOVA test for the degree of polymerisation (*Y*3) by augmented RSM.
