# Supplementary material for: Process Optimisation of Ultrasound-Assisted Extraction of Oligosaccharides from Coconut Husk
Source: ScientificWorldJournal. 2023 Apr 15;2023:9427831. doi: 10.1155/2023/9427831 (PMC10122602; doi:10.1155/2023/9427831)
Supplement: Supplementary Materials — Figure S1. Normality plot of residuals before transformation. Table S1. ANOVA test for total carbohydrates (Y1) by two-level fractional factorial design. Table S2. ANOVA test for total carbohydrates (Y1) by augmented RSM. Table S3. ANOVA test for total reducing sugar (Y2) by two-level fractional factorial design. Table S4. ANOVA test for the degree of polymerisation (Y3) by two-level fractional factorial design. Table S5. ANOVA test for the degree of polymerisation (Y3) by augmented RSM. [file 9427831.f1.pdf]

Figure S1: Normality plot of residuals before transformation.

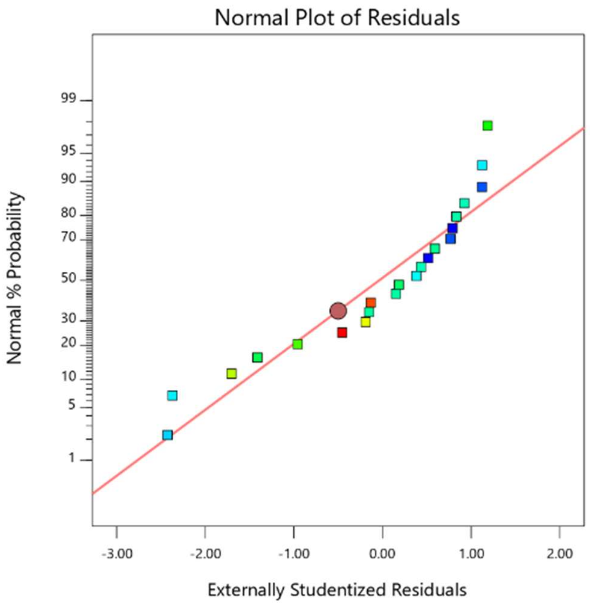

Table S1: ANOVA test for total carbohydrates (Y_1) by two-level fractional factorial design.

Source	Sum of Squares	*df*	Mean square	*F* value	*p*-value Prob>F
Block	7.406×10^{-6}	1	7.406×10^{-6}		
Model	0.0336	4	0.0084	28.03	< 0.0001*
X_2	0.0017	1	0.0017	5.54	0.0326*
X_4	0.0199	1	0.0199	66.54	< 0.0001*
X_5	0.0081	1	0.0081	26.97	0.0001*
X_2X_4	0.0039	1	0.0039	13.06	0.0025*
Curvature	0.0032	1	0.0032	10.76	0.0051*
Residual	0.0045	15	0.0003		
Lack of Fit	0.0032	11	0.0003	0.9404	0.5803
Pure Error	0.0013	4	0.0003		
Cor Total	0.0413	21			

X_2, extraction time (min); X_4, sodium hydroxide concentration (%w/v); X_5, solid-to-liquid ratio; *df*: degrees of freedom. *significant different ($p < 0.05$)

Table S2: ANOVA test for total carbohydrates (Y_1) by augmented RSM.

Source	Sum of Squares	*df*	Mean Square	*F*-value	*p*-value Prob > F
Block	0.0034	2	0.0017		
Model	0.0450	9	0.0050	16.77	< 0.0001*
X_1	0.0022	1	0.0022	7.22	0.0135*
X_2	0.0018	1	0.0018	6.00	0.0228*
X_3	9.483×10^{-6}	1	9.483×10^{-6}	0.0318	0.8601
X_4	0.0225	1	0.0225	75.60	< 0.0001*
X_5	0.0078	1	0.0078	26.07	< 0.0001*
X_1X_3	0.0018	1	0.0018	6.00	0.0227*
X_2X_4	0.0027	1	0.0027	9.19	0.0061*
X_3X_4	0.0013	1	0.0013	4.37	0.0484*
X_2^2	0.0049	1	0.0049	16.41	0.0005*
Residual	0.0066	22	0.0003		
Lack of Fit	0.0053	16	0.0003	1.58	0.2973
Pure Error	0.0013	6	0.0002		
Cor Total	0.0550	33			

X_1, extraction temperature (°C); X_2, extraction time (min); X_3, ultrasonicator power (watt); X_4, sodium hydroxide concentration (%w/v); X_5, solid-to-liquid ratio. *Significant different ($p <$ 0.05).

Table S3: ANOVA test for total reducing sugar (Y_2) by two-level fractional factorial design.

Source	Sum of Squares	*df*	Mean Square	*F*-value	*p*-value Prob> F
Block	0.0018	1	0.0018		
Model	0.1354	4	0.0338	25.46	< 0.0001*
X_2	0.0197	1	0.0197	14.85	0.0016*
X_5	0.0986	1	0.0986	74.21	< 0.0001*
X_3X_4	0.0065	1	0.0065	4.91	0.0426*
$X_1X_3X_4$	0.0105	1	0.0105	7.87	0.0133*
Curvature	0.0014	1	0.0014	1.06	0.3191
Residual	0.0199	15	0.0013		
Lack of Fit	0.0148	11	0.0013	1.05	0.5313
Pure Error	0.0051	4	0.0013		
Cor Total	0.1585	21			

X_1, extraction temperature (°C); X_2, extraction time (min); X_3, ultrasonicator power (watt); X_4, sodium hydroxide concentration (%w/v); X_5, solid-to-liquid ratio. *Significant different ($p <$ 0.05).

Table S4: ANOVA test for degree of polymerisation (Y_3) by two-level fractional factorial design.

Source	Sum of Squares	*df*	Mean Square	*F*-value	p-value (Prob>F)
Block	0.0062	1	0.0062		
Model	0.4410	5	0.0882	12.54	< 0.0001*
X_2	0.0422	1	0.0422	6.00	0.0281*
X_4	0.1752	1	0.1752	24.91	0.0002*
X_5	0.1178	1	0.1178	16.75	0.0011*
X_2X_4	0.0387	1	0.0387	5.51	0.0342*
X_4X_5	0.0672	1	0.0672	9.55	0.0080*
Curvature	0.0808	1	0.0808	11.50	0.0044*
Residual	0.0984	14	0.0070		
Lack of Fit	0.0695	10	0.0069	0.9587	0.5677
Pure Error	0.0290	4	0.0072		
Cor Total	0.6265	21			

X_1, extraction temperature (°C); X_2, extraction time (min); X_3, ultrasonicator power (watt); X_4, sodium hydroxide concentration (%w/v); X_5, solid-to-liquid ratio. *Significant different ($p < 0.05$)

Table S5: ANOVA test for degree of polymerisation (Y_3) by augmented RSM.

Source	Sum of Squares	*df*	Mean Square	*F*-value	p-value
Block	0.0069	2	0.0035		
Model	0.0465	6	0.0078	11.86	< 0.0001*
X_2	0.0038	1	0.0038	5.82	0.0235*
X_4	0.0176	1	0.0176	27.00	< 0.0001*
X_5	0.0154	1	0.0154	23.49	< 0.0001
X_2X_4	0.0061	1	0.0061	9.35	0.0053*
X_3X_5	0.0029	1	0.0029	4.38	0.0467*
X_3^2	0.0064	1	0.0064	9.72	0.0045*
Residual	0.0163	25	0.0007		
Lack of Fit	0.0113	19	0.0006	0.7130	0.7354
Pure Error	0.0050	6	0.0008		
Cor Total	0.0698	33			

X_1, extraction temperature (°C); X_2, extraction time (min); X_3, ultrasonicator power (watt); X_4, sodium hydroxide concentration (%w/v); X_5, solid-to-liquid ratio. *Significant different ($p <$ 0.05).
